# The Longitudinal Effects of Resisted and Assisted Sprint Training on Sprint Kinematics, Acceleration, and Maximum Velocity: A Systematic Review and Meta-analysis

**DOI:** 10.1186/s40798-024-00777-7

**Published:** 2024-10-11

**Authors:** Simen Myrvang, Roland van den Tillaar

**Affiliations:** Nord University, Levanger, Norway

**Keywords:** Sprint Mechanics, Contact and Flight time

## Abstract

**Background:**

Sprinting is important for both individual and team sports, and enhancing performance is often done through resisted, assisted, or combined sprint training. However, the effectiveness of these methods compared to traditional sprint training remains inconclusive. The objective of this review with meta-analysis was to review the current literature on intervention studies analyzing the effects of resisted, assisted, and combined (resisted–assisted) training on sprint kinematics and performance in terms of acceleration and maximum velocity.

**Methods:**

A literature search was conducted using SPORTDiscus up to and including April 19, 2023. The following eligibility criteria were applied: (1) a longitudinal study over a minimum of four weeks; (2) studies using resistance (sleds, parachutes, uphill slope, towing devices) or assistance (towing devices, downhill slope), or a combination of both; (3) a main intervention focused on resisted or assisted training, or a combination of both; (4) measurement of maximum velocity, acceleration measured in (s) with a minimum distance of 10-m, or kinematic changes such as step frequency, ground contact time, flight time, and step length; and (5) peer-reviewed studies.

**Results:**

Twenty-one studies were included in this review with meta-analysis. Kinematic changes, changes in acceleration, and changes in maximum velocity were analyzed. Only resisted sprint training was associated with a significant improvement in 10-m acceleration compared to normal (i.e. without assistance or resistance) sprinting (Z = 2.01, *P* = 0.04). With resisted, assisted and combined sprint training no significant changes in kinematics, 20-m times or maximum velocity were found when compared to normal sprint training. However, in the within group, effect sizes resisted sprint training had a moderate effect on 10-m times. A moderate effect on ground contact time, step frequency, 10-and 20-meter time after assisted sprint training was found, while combined sprint training had a moderate effect on maximum velocity.

**Conclusion:**

Resisted sprint training seems to be effective for improving acceleration ability, with significant decreases in the 10-m times. There were no other significant findings, suggesting that normal sprinting yields the same change in 20-m times, kinematics and maximum velocity as resisted, assisted and combined sprint training. However, moderate effect sizes using these different training methods were found, which may suggest that the different training forms could be useful for improving different parts of the sprint and changing the kinematics. Combination (uphill–downhill) sprint training seems to be effective at improving maximum velocity, while assisted sprint training was the most effective training to increase step frequency, which can affect sprint performance positively. However, more studies, especially in assisted sprints, need to be conducted to determine the full effect of these training forms.

**Supplementary Information:**

The online version contains supplementary material available at 10.1186/s40798-024-00777-7.

## Key Points

• Resisted sprint training is effective for improving acceleration ability; however, combination (uphill–downhill) sprint training is more effective at improving maximum velocity.

• The overall group analysis shows that all of the training forms decrease the flight time.

• Assisted sprint training is the most effective training to increase step frequency, which can affect sprint performance positively.

**Supplementary Information:**

The online version contains supplementary material available at 10.1186/s40798-024-00777-7.

## Background

Sprinting is important for many different sports. In track and field, there are four main sprint distances, in which the main goal is to sprint as fast as possible (60 m, 100 m, 200 m, and 400 m). However, sprinting is also key in many other disciplines, such as pole vault, long jump, triple jump, and javelin. There are strong relationships found between maximum velocity with pole vault performance and javelin throw performance, and between 30-m sprints and long jump performance [1–3]. This is an indication that sprinting is of significant importance whether in pure sprinting or other disciplines in track and field.

Sprint performance extends its importance to team sports as well. Team-handball, characterized by rapid transitions between attacking and defensive play on a relatively small court, demands swift movements from players. Due to the small court, players will not have time to reach maximum velocity during the run to the other side. Therefore, it would be relevant for handball players to focus on increasing their acceleration ability as emphasized by Luteberget et al. [4]. However, research suggests that improving maximum velocity could have a positive impact on acceleration [5], making maximum velocity training relevant even though the court is small. In soccer and rugby, the need for high maximum velocity as well as good acceleration is critical when avoiding opponents or winning possession [6, 7]. Unlike handball, the larger playing fields in these sports provide players with sufficient time to reach their maximum velocity. The importance of acceleration is also huge, as in these sports players must react quickly to changes in direction, etc. Athletes with superior acceleration and maximum velocity will generally have an advantage in soccer, rugby and field hockey as faster athletes will be able to reposition themselves more quickly when contesting for the ball and in scoring opportunities [8–10].

### Different Training Methods

Improving sprint performance primarily involves specific sprint training. According to Rumpf et al. [8], specific sprint training consists of (1) sprinting without any loading on a flat surface, normal sprinting; (2) resisted sprinting, by sleds, bands, uphill running, or parachutes; and (3) assisted sprinting, by a towing system or a downhill slope. Another specific sprint training method is a combination of both resisted (uphill) and assisted (downhill) sprints, used mainly by Paradisis et al. [11]. Sprinters need to develop specific attributes during their training, such as improved acceleration or a higher maximum velocity. Resisted sprint training aims to improve horizontal force production and can be considered a type of specific-strength training for sprinting. Specific-strength training adheres to the specificity principle, meaning that exercises closely resembling the desired skill yield better learning transfer. In the context of sprinting, specific strength can be developed through resisted sprint training [12]. Both resisted, normal sprinting and resisted sprinting share similar motor patterns and neuromuscular traits, facilitating a seamless skill transfer [13].

Using assisted sprint training it is theorized that athletes can break their speed barrier, which is the limit of velocity the athlete can reach on their own [14]. With continuous and progressive overspeed stimulus over time, this can improve sprint performance and move the speed barrier [15]. Sprinters also do non-specific training like plyometric and strength training to improve sprint performance [16, 17], but that is not the scope of this review.

### Acceleration Training

Acceleration training in the track and field community is performed using starting blocks from a crouched position or a three-point start position. The distances over which people train vary, and better sprinters reach higher top speeds and accelerate longer than their lower-performing counterparts [17]. Another way of increasing acceleration may be resisted sprint training using a sled, parachute, or band. Acute studies showed changes in running kinematics that may enhance acceleration ability, like increased ground contact time and/or increased step frequency [18, 19]. With increased ground contact time, athletes will be able to apply more horizontal force to each step if applied in the correct direction. An increase in step frequency would produce more steps with increased ground contact time; therefore, they will produce more force in the acceleration during resisted sprints.

Resisted sprint training has also shown a positive acute effect in the normal 20-m sprint performance (2%) after the application of one resisted run-in for team handball players, which is an indication that the use of resisted sprints in sprint training could have a positive effect on normal sprints. However, with several resisted runs, it caused fatigue, decreased vertical stiffness, step length, and frequency [20] and it is not known what the longitudinal effects of this type of training are.

Resisted sprint training is commonly used in the track and field community, where it is widely accepted as a good way to improve acceleration [18]; however, its use for this reason also growing in team sports such as soccer, handball, and rugby [4, 21–23]. Even though it is frequently used, its effect compared to normal sprinting remains unclear [17], and will be analyzed further in this review.

### Maximum Velocity Training

Maximum velocity training is usually performed using flying sprints, where the athlete has a build-up distance (e.g. 20 m) to reach maximal velocity, followed by a distance in which the athlete tries to maintain maximal velocity as long as possible without a decrease [17]. Training using assistance may be a method of increasing maximum velocity. Assisted sprint training is sprinting while being pulled, being towed, or running downhill. With assisted sprint training, athletes train with a higher running velocity than their body can produce on its own. Assisted sprint training is not widely used, but acute studies show some indications of possible changes [20, 24, 25]. When running with assistance, it is found that ground contact time reduces and the flight time increases [20], which could be of importance, especially at maximal velocity where ground contact time is a performance factor [26]. Acute studies also showed changes in kinematics with increased step length, increased frequency, as well as increased velocity at every step when compared to a normal sprint [20, 24, 25]. As speed is a product of step length and step frequency, and an increase in one of these two variables, or both, this will result in a higher running speed [27]. However, it is not clear if these acute changes over a longer training period of assisted sprints will also transfer positively to normal sprint performance.

### Could Athletes Develop both Acceleration and Maximal Velocity at the Same Time?

Another form of sprint-specific training is a method that involves combining resisted, unresisted, and assisted sprint training in a single run. This has been done using an uphill–downhill installation. When resistance is applied during the acceleration phase, acute kinematic changes occur, including increased ground contact time, reduced flight time, and increased step frequency, as well as greater flexion in the knee, hip, and ankle angles and increased trunk lean during the early acceleration [20, 28, 29]. It could be expected that kinematic changes would also apply to uphill sprinting, but it is unclear if the same changes occurring with resistance caused by towing occur when running uphill.

With the uphill–downhill system [30], the uphill section will increase sprint resistance. The athletes would not reach their maximum velocity in the uphill section but keep on accelerating until they reach the horizontal part. On the horizontal part of the uphill-downhill system the athletes are at the last part of the acceleration phase and the start of the maximum velocity phase, so they will have a normal start to their maximum velocity phase. With the downhill during the maximum velocity phase, the athletes will achieve greater speed towards the end of the maximum velocity phase of the run and re-accelerate in the downhill section. They have prolonged time at their maximum velocity. It could be assumed that the same acute kinematic changes seen with assisted sprint training would apply downhill: increased flight time, shorter ground contact time, and increased stride length [11, 20, 24, 25, 30], but as there are no current studies comparing kinematic changes when sprinting downhill to assisted sprint runs, it remains unclear if these acute effects would be the same.

The acute effects of specific sprint training methods such as resisted training, assisted training, and a combination of both have shown alterations in sprint kinematics and the ability to improve acceleration and/or maximum velocity [11, 20, 24, 25]. However, not much is known about how these acute effects impact sprint performance over time. It would be beneficial to determine the longitudinal effects of these different training methods, as well as compare the effects of resisted training and assisted training to normal sprinting. Therefore, the objective of this systematic review with meta-analysis is to review the state of the current literature through studies that have analyzed the longitudinal effects of normal sprinting, resisted, assisted, and combined (uphill–downhill) sprint training on sprint kinematics, sprint acceleration, and maximum velocity.

## Methods

### Literature Search

The systematic review process was conducted according to the PRISMA guidelines and checklist [31]. Keywords and Boolean operators were used in the research process, and the systematic review of the literature was performed using SPORTDiscus, PubMed and Web of Science. The search results were limited to studies published up to April 19, 2023. Two separate searches were conducted: one for resisted sprint training and one for assisted sprint training.

For the resisted sprint search, the following keywords and Boolean phrases were used: « Resisted sprint training » OR « Resisted sprint » OR « Resisted sled training » OR « Resisted sled » OR « Sled resisted sprint training ».

For the assisted sprint search, the following keywords and Boolean phrases were used: « Assisted sprint » OR « Assisted sprint training » OR “Overspeed training”.

The results were limited to articles in English. Further records were added based on citations from other articles and studies already known.

### Inclusion Criteria

The criteria set for this review were as follows: (1) The longitudinal studies had to be conducted over a minimum of four weeks. (2) The studies must have used some resistance in the form of a sled, a parachute, an uphill slope, or a towing device OR some assistance in the form of a towing device or a downhill slope. Also, studies were included that combined both training forms and compared those two training forms. (3) The main intervention in the studies had to be a resisted training form, assisted training form, or a combination of both. (4) The included studies must measure maximum velocity, acceleration measured in (s) with a minimum distance of 10-m, or kinematic changes (i.e., step frequency, ground contact time, flight time, and step length). (5) The studies included had to be peer-reviewed. The studies were not differentiated based on sex and training history, as there are only a few studies published on the longitudinal effects of these training methods. One study without a control group was included [32]. This study had three different groups with different loads. This study was not eligible for the meta-analysis, but was relevant for the within group ES (Table 1).

**Table 1:** Training frequency, volume, surface load, and measuring tools of the interventions. BW = Body weight

Study	Sessions per week	Session volume (m)	Surface	Load	Control group	Measurement
Bachero-Mena et al. [34]	2	100–210	Track	Low: 5% BW Medium: 12.5% BW High: 20%BW	-	Photocell
Cahill et al. [36]	2	90–420	Hard surface	25% velocity decrement 50% velocity decrement 75% velocity decrement	Normal sprinting	Radar device
Cahill et al. [37]	2	90–420	Hard surface	25% velocity decrement 50% velocity decrement 75% velocity decrement	Normal sprinting	Radar device
Clark et al. [39]	2	240–400	Hard surface	10% BW sled 18.5% BW vest	Normal sprinting	Photocell
Hicks et al. [40]	2	60–120	Hard surface	97.5 *N* ± 15 N	Normal sprinting	Radar gun
Kristensen et al. [15]	3	110	Hard surface	Assisted: 6% faster than normal sprinting Resisted: 6% velocity decrement	Normal sprinting	Photocell
Lockie et al. [41]	2	195–320	Grass	12.6% BW	Normal sprinting	Velocimeter with Stopwatch
Luteberget et al. [4]	2	240–280	Hard surface	12.4% BW	Normal sprinting	Photocell
Makaruk et al. [42]	2	All distances with a 20-m run-in 80–120	Track	4% faster than maximal velocity	Normal sprinting	Photocell
Martinopoulou et al. [42]	3	120–200	Track	Medium parachute	Normal sprinting	Photocell
Morin et al. [21]	2	100		80% BW	Normal sprinting	Indirect method
Paradisis et al. [44]	3	480	Track	3 degrees uphill, 3 degrees downhill	Normal sprinting	Photocell
Paradisis et al. [11]	3	480–800	Track	3 degrees uphill, 3 degrees downhill	Normal sprinting	Photocell
Paradisis et al. [30]	3	480–800	Track	3 degrees uphill, 3 degrees downhill	Normal sprinting	Photocell
Pareja-Blanco et al. [44]	1	120–240	Track	40% BW	Normal sprinting	Photocell
Rodríguez-Rosell et al. [45]	1	180	Track	20% BW 40% BW 60% BW 80% BW	Normal sprinting	Photocell
Sinclair et al. [46]	2	180		20% MV decrement	Normal sprinting	Photocell
Spinks et al. [22]	2	215–340	Hard surface	10% maximal velocity decrement	Normal sprinting	Stopwatch
Upton [23]	3	137	Soccer field	14.7% BM assisted 12.6% BW resisted	Normal sprinting	Infrared beam timing system
West et al. [47]	2	60	Hard surface	12.6% BW	Normal sprinting	Photocell
Zafeiridis et al. [48]	3	280	Track	5 kg sled	Normal sprinting	Photocell

BW = Body weight

### Study Selection and Data Extraction

Titles and abstracts of the studies in the literature search were evaluated. For this review, only studies that met the inclusion criteria were used. If the abstract showed potential, the full text of the article was read to determine whether it met the inclusion criteria stated in the previous section. Also, studies were included that measured step frequency, step length, ground contact time, and flight time, as these variables showed changes in acute studies. Furthermore, the studies included had to be categorized into resisted, assisted and combined sprint training. Furthermore, the studies’ quality was assessed using the PEDro scale [33]. The PEDro score had to be 5 or better to be included in this review and meta-analysis (see supplementary file). Data on sample size, duration of the interventions, amount of female participation, training load, frequency, age, weight, height, and training status of the subjects were extracted. If the mean and standard deviation could not be obtained from published records, the corresponding authors were contacted via e-mail [32].

### Statistical Analysis

Review Manager Software (RevMan 5.4, Cochrane Collaboration, Oxford, UK) was used for the meta-analysis, which provided a randomized effect model for the included studies. The randomized effect model was chosen, because it accounts for variability both within and between studies, providing a more generalizable estimate of the effect size. The statistical analyses included intervention means, control means, standard deviations, and sample sizes for each study. The measurements for the 10- and 20-m times were taken by measuring the time (s), maximum velocity was measured in speed (m/s), step length was measured in m, contact time and flight time was measured in s, and step frequency measured in Hz.

**Fig. 1:**
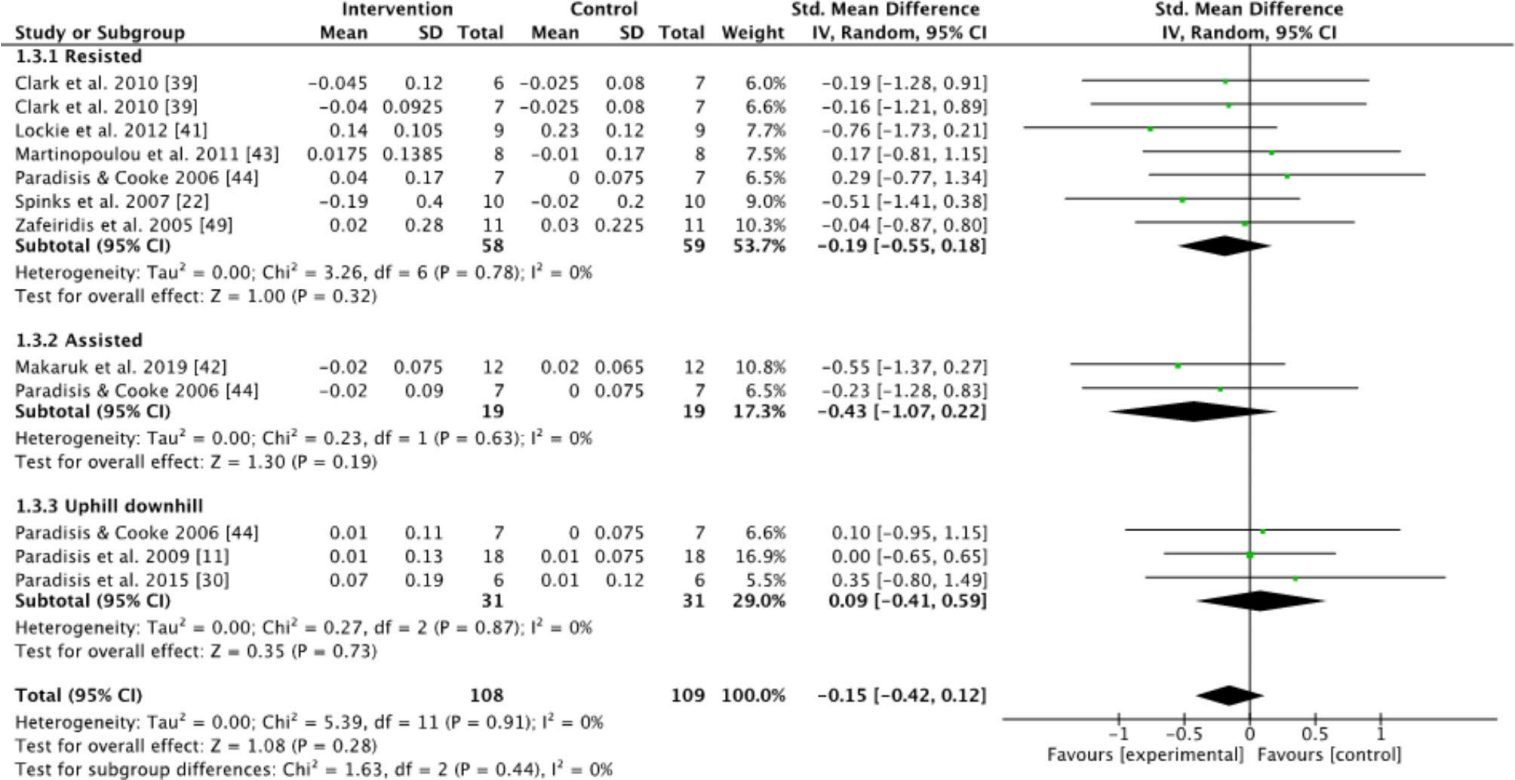
Standardized mean difference between the pre- and post-test on step length (m) for the intervention and control groups. Squares represent mean difference for each trial. Diamonds represent the pooled mean difference across trials

**Fig. 2:**
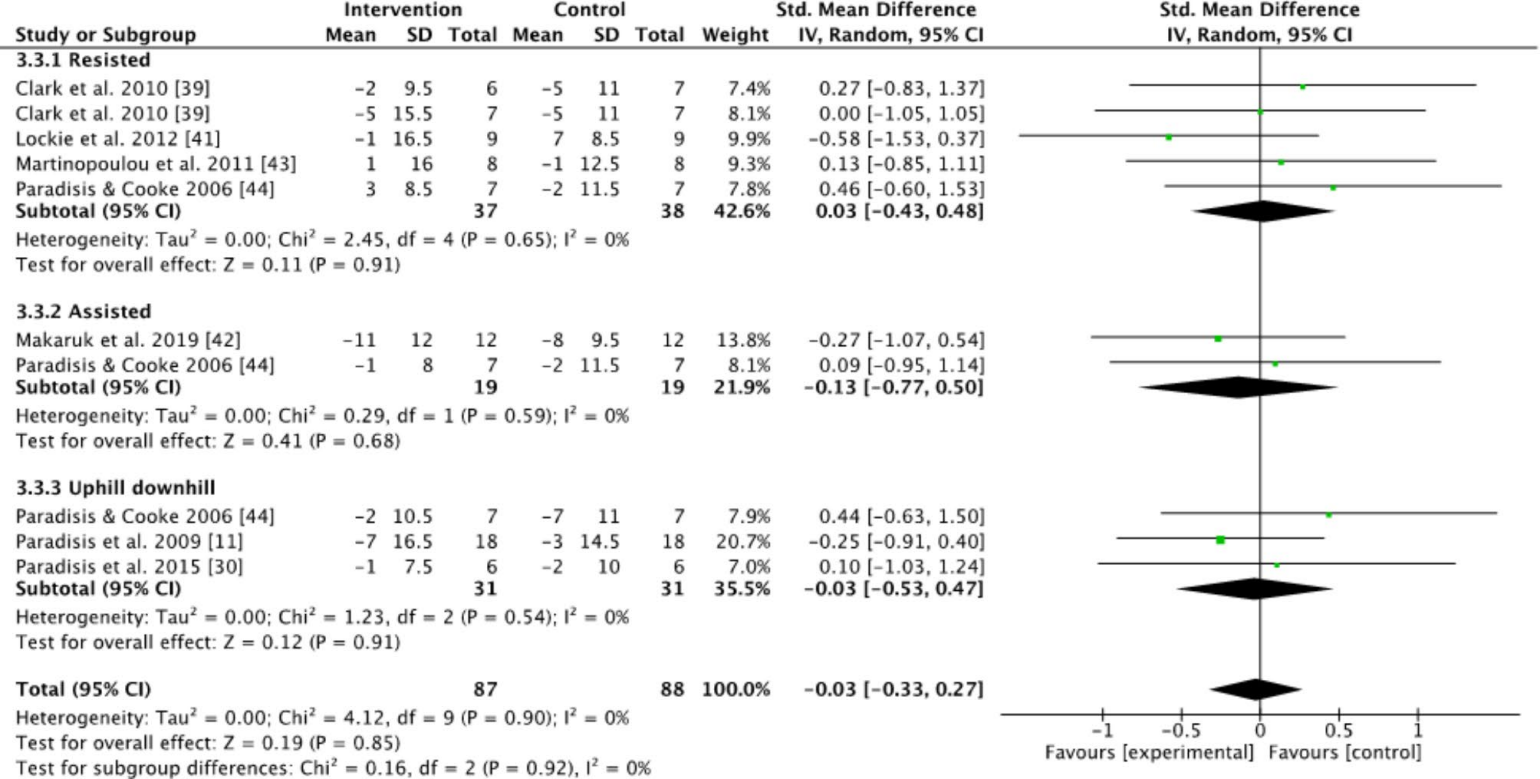
Standardized mean difference between the pre- and post-test on contact time (s) for the intervention and control groups. Squares represent mean difference for each trial. Diamonds represent the pooled mean difference across trials

**Fig. 3:**
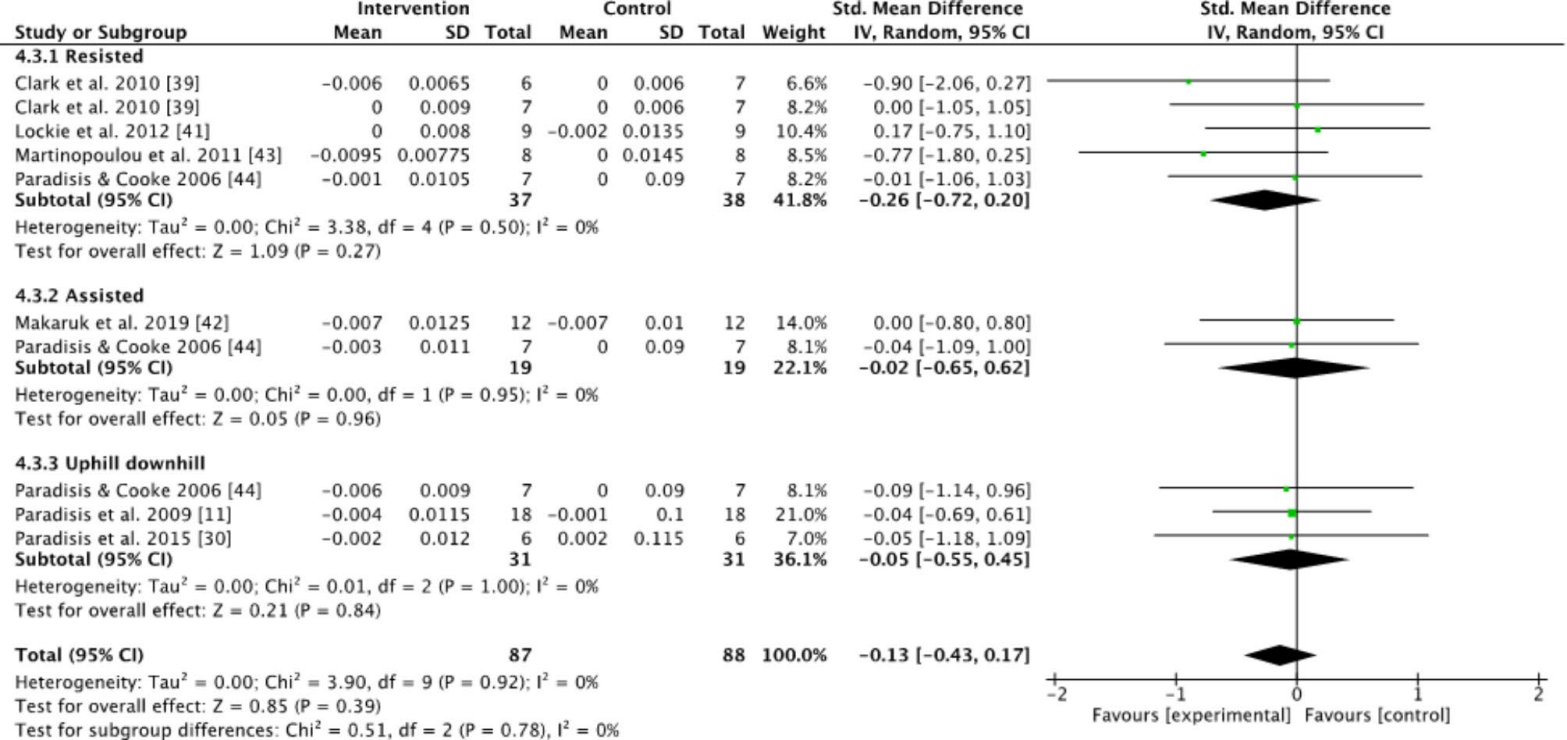
Standardized mean difference between the pre- and post-test on flight time (s) for the intervention and control groups. Squares represent mean difference for each trial. Diamonds represent the pooled mean difference across trials

**Fig. 4:**
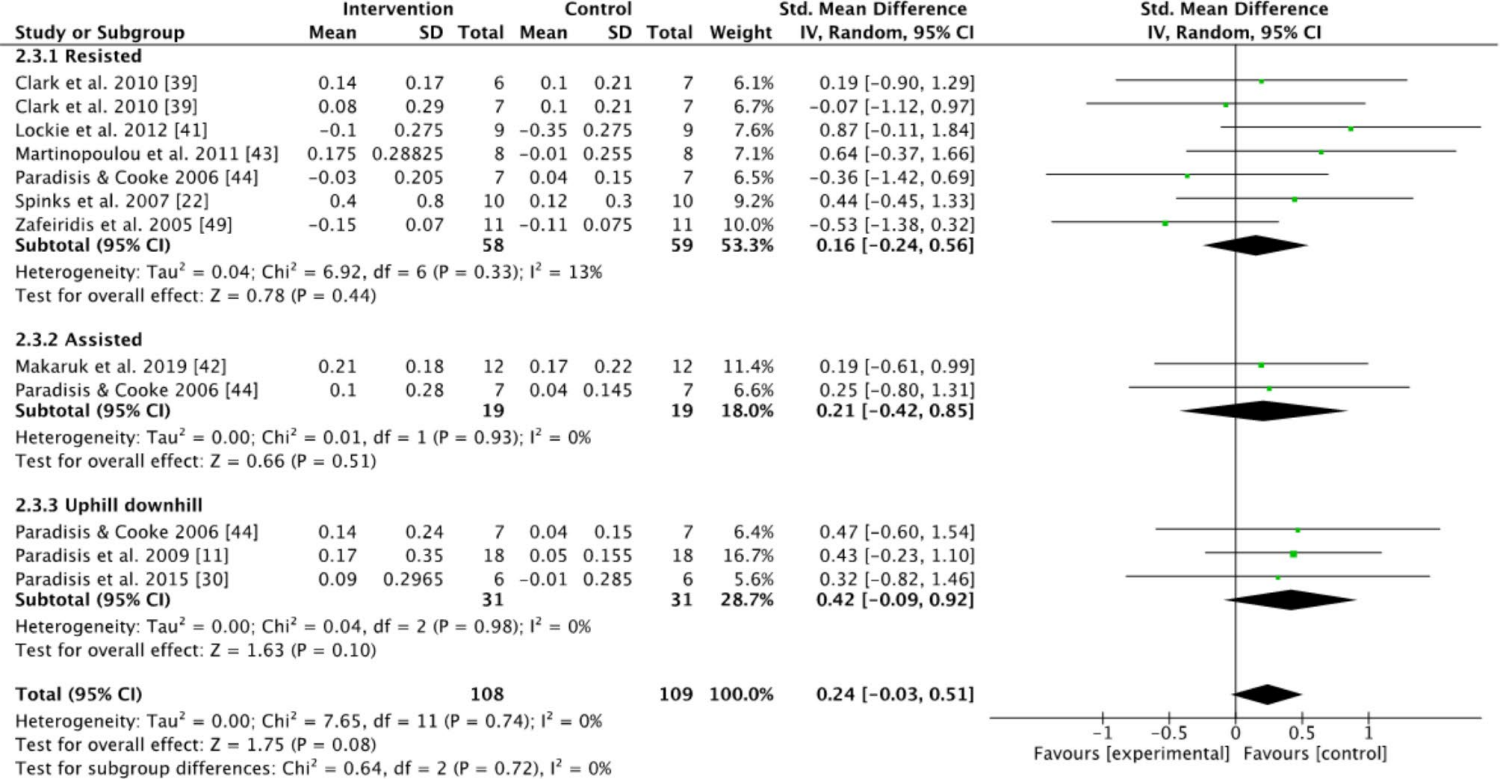
Standardized mean difference between the pre- and post-test on step frequency (Hz) for the intervention and control groups. Squares represent mean difference for each trial. Diamonds represent the pooled mean difference across trials

**Fig. 5:**
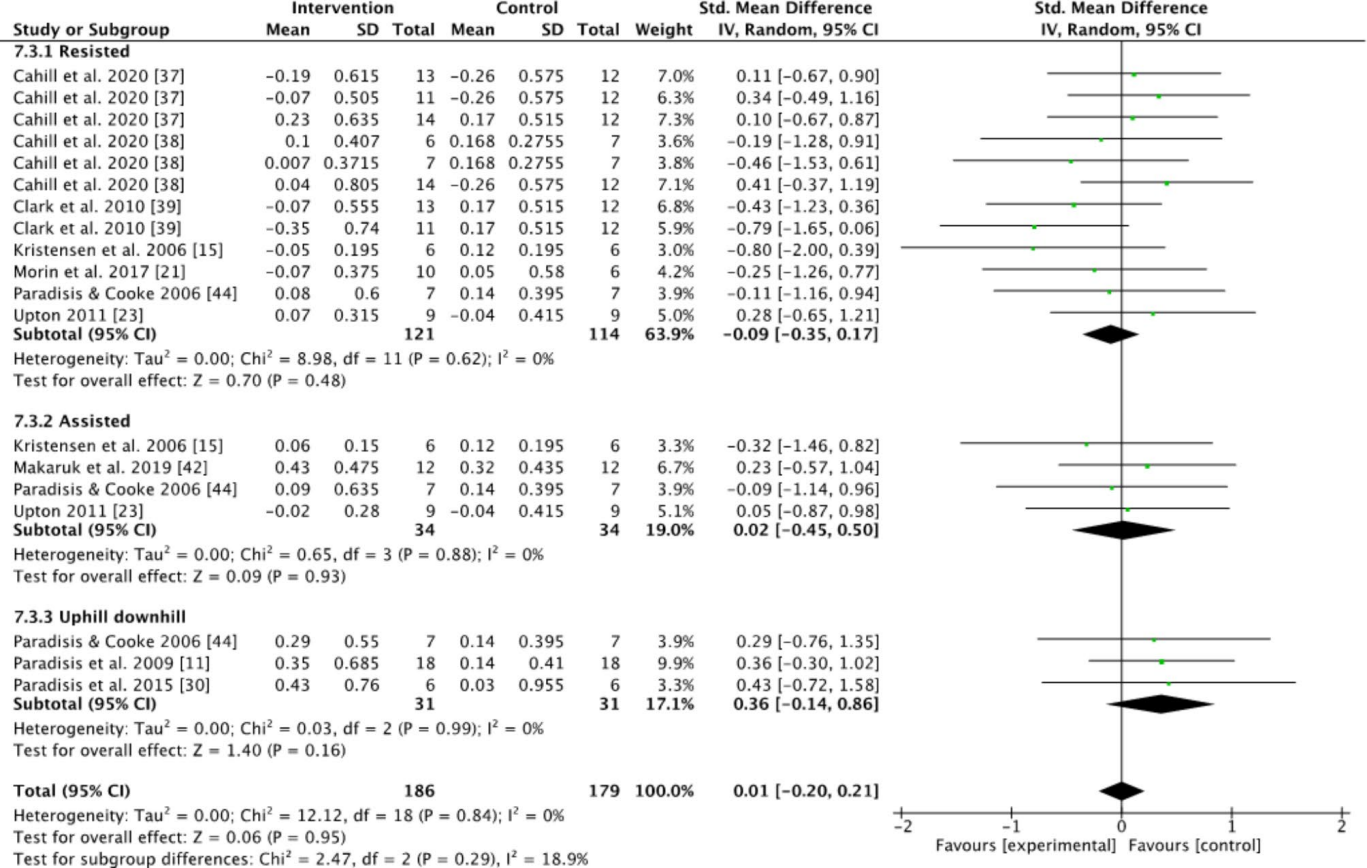
Standardized mean difference between the pre- and post-test for maximum velocity (m/s) for the intervention and control groups. Squares represent mean difference for each trial. Diamonds represent the pooled mean difference across trials

**Fig. 6:**
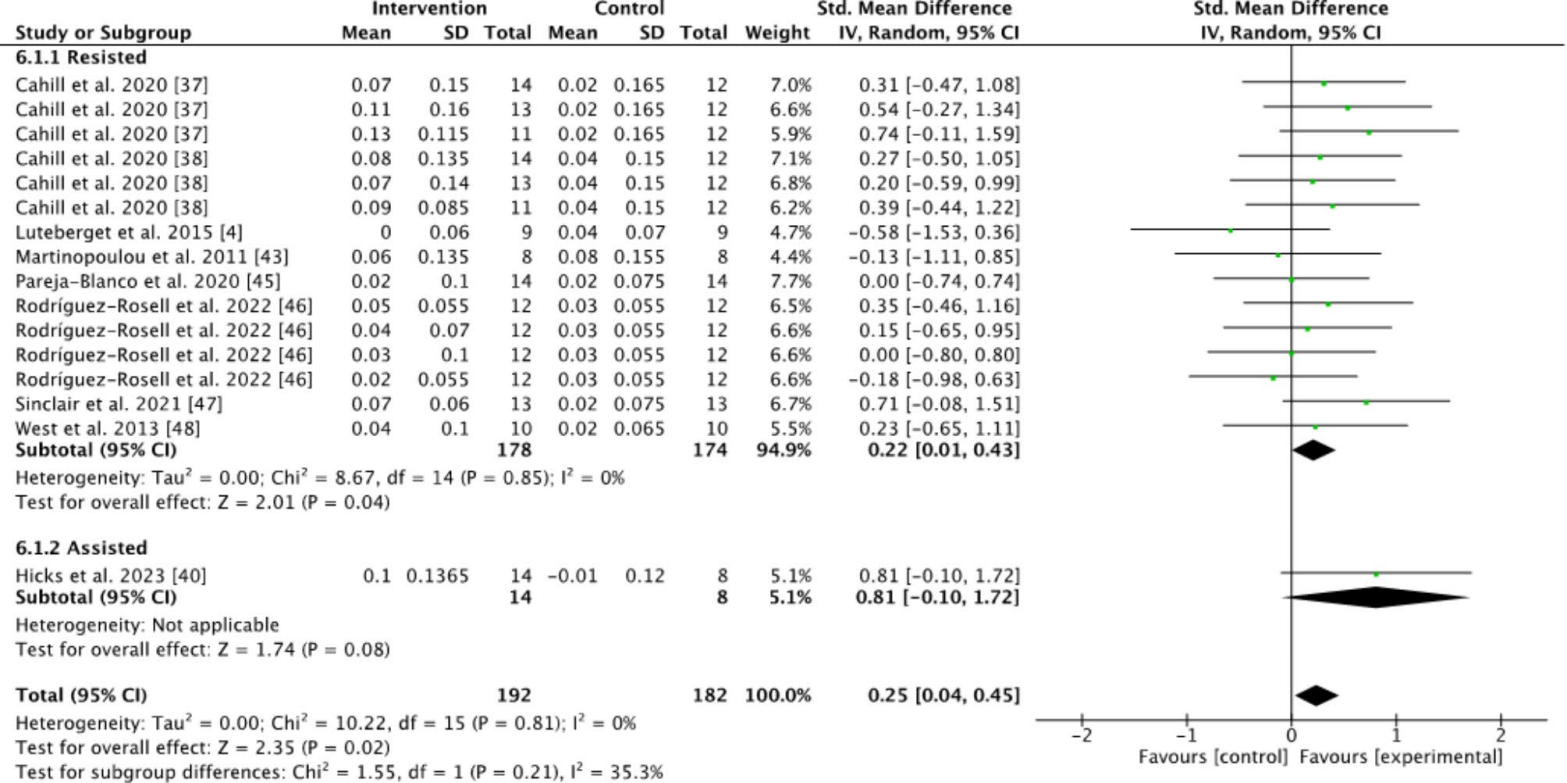
Standardized mean difference between the pre- and post-test 10-m times for the intervention and control groups. Squares represent mean difference for each trial. Diamonds represent the pooled mean difference across trials

**Fig. 7:**
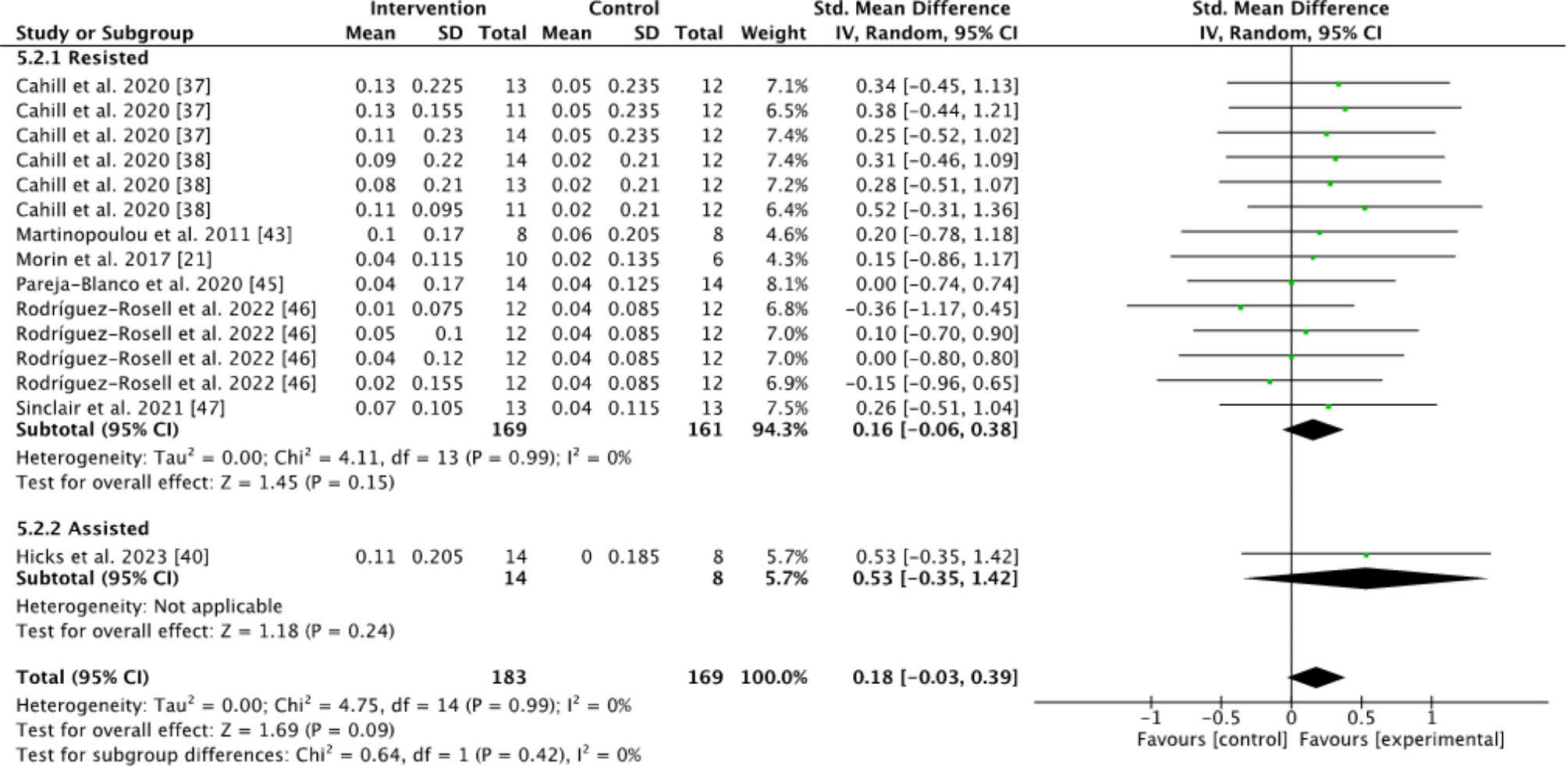
Standardized mean difference between the pre- and post-test 20-m times for the intervention and control groups. Squares represent mean difference for each trial. Diamonds represent the pooled mean difference across trials

Subgroups dividing resisted sprint training, assisted sprint training, and combined training were used to compare the kinematic changes and changes in maximum velocity between the pre- and post-tests for the intervention group. In the analyses for kinematics and maximum velocity, favoring experimental means a greater positive change in performance (e.g., increase in maximum velocity or beneficial kinematic adaptation) compared to the control group. Conversely, favoring control means the control group showed a greater positive change relative to the experimental group (Figs.  1, 2, 3, 4 and 5). For the 10-m and 20-m analyses, favoring the experimental group indicated an improvement in acceleration compared to the control group, whereas favoring the control group indicated no improvement in acceleration relative to the experimental group (Figs. 6 and 7). The effect size (ES) used in this review and Table 2 is Cohen’s d. The interpretation of Cohen’s d ESs was as follows: small (> 0.2), moderate (> 0.5), and large (> 0.8) [34].

**Table 2:** Within group effects for the different measurements calculated using Cohen´s d

Study	Step length	Contact time	Flight time	Step frequency	10 m	20 m	Maximum velocity
**Resisted**							
Bachero-Mena et al. [34]							
Low load					0.46	0.38	
Medium load					0.18	0.24	
High load					0.20	0.43	
Cahill et al. [36]							
25% velocity decrement					0.59	0.41	0.05
50% velocity decrement					0.50	0.38	0.31
75% velocity decrement					1.06	1.16	0.14
Cahill et al. [37]							
25% velocity decrement					0.47	0.48	0.36
50% velocity decrement					0.69	0.58	0.13
75% velocity decrement					1.13	0.84	0.47
Clark et al. [38]							
Sled	0.43	0.32	0	0.28			0.02
Vest	0.38	0.21	0.92	0.82			0.24
Kristensen et al. [15]							0.26
Lockie et al. [41]	1.33	0.06	0	0.36			
Luteberget et al. [4]					0		
Martinopoulou et al. [42]	0.13	0.06	1.22	0.6	0.44	0.59	
Morin et al. [21]						0.35	0.19
Paradisis et al. [44]							
Uphill	0.24	0.35	0.09	0.15			0.13
Pareja-Blanco et al. [44]					0.20	0.24	
Rodríguez-Rosell et al. [45]							
20%					0.36	0.13	
40%					0.91	0.50	
60%					0.57	0.33	
80%					0.3	0.13	
Sinclair et al. [46]					1.16	0.67	
Spinks et al. [22]	0.48			0.5			
Upton [23]							0.22
West et al. [47]					0.4		
Zafeiridis et al. [48]	0.07			2.14			
**Combined ES**	0.04	0.06	0.40	0.25	0.55	0.46	0.04
**Assisted**							
Hicks et al. [40]					0.73	0.53	
Kristensen et al. [15]							0.40
Makaruk et al. [42]	0.27	0.91	0.56	1.17			0.90
Paradisis et al. [44]	0.22	0.12	0.27	0.36			0.14
Upton [23]							0.07
**Combined ES**	0.24	0.6	0.43	0.67	0.73	0.53	0.36
**Uphill + Downhill**							
Paradisis et al. [44]							
Uphill + downhill	0.09	0.19	0.67	0.58			0.53
Paradisis et al. [11]	0.08	0.42	0.34	0.49			0.51
Paradisis et al. [30]	0.37	0.13	0.17	0.30			0.56
**Combined ES**	0.21	0.29	0.37	0.45			0.54

### Publication Bias

Publication bias was addressed by the funnel plots in Review Manager Software. As suggested by Higgins et al. [35] a trim and fill method was performed on analyses that had 10 or more studies included in each group. The trim and fill method was performed using R with the metafor package (R: A language and environment for statistical computing, R core team, Vienna, Austria). Due to the limited number of studies in the different subgroups on sprint kinematics, it was not possible to perform an accurate trim and fill on these subgroups. Publication bias was therefore only addressed in the 10-m analysis, 20-m analysis and the maximum velocity analysis.

## Results

### Characteristics of the Included Studies

**Fig. 8:**
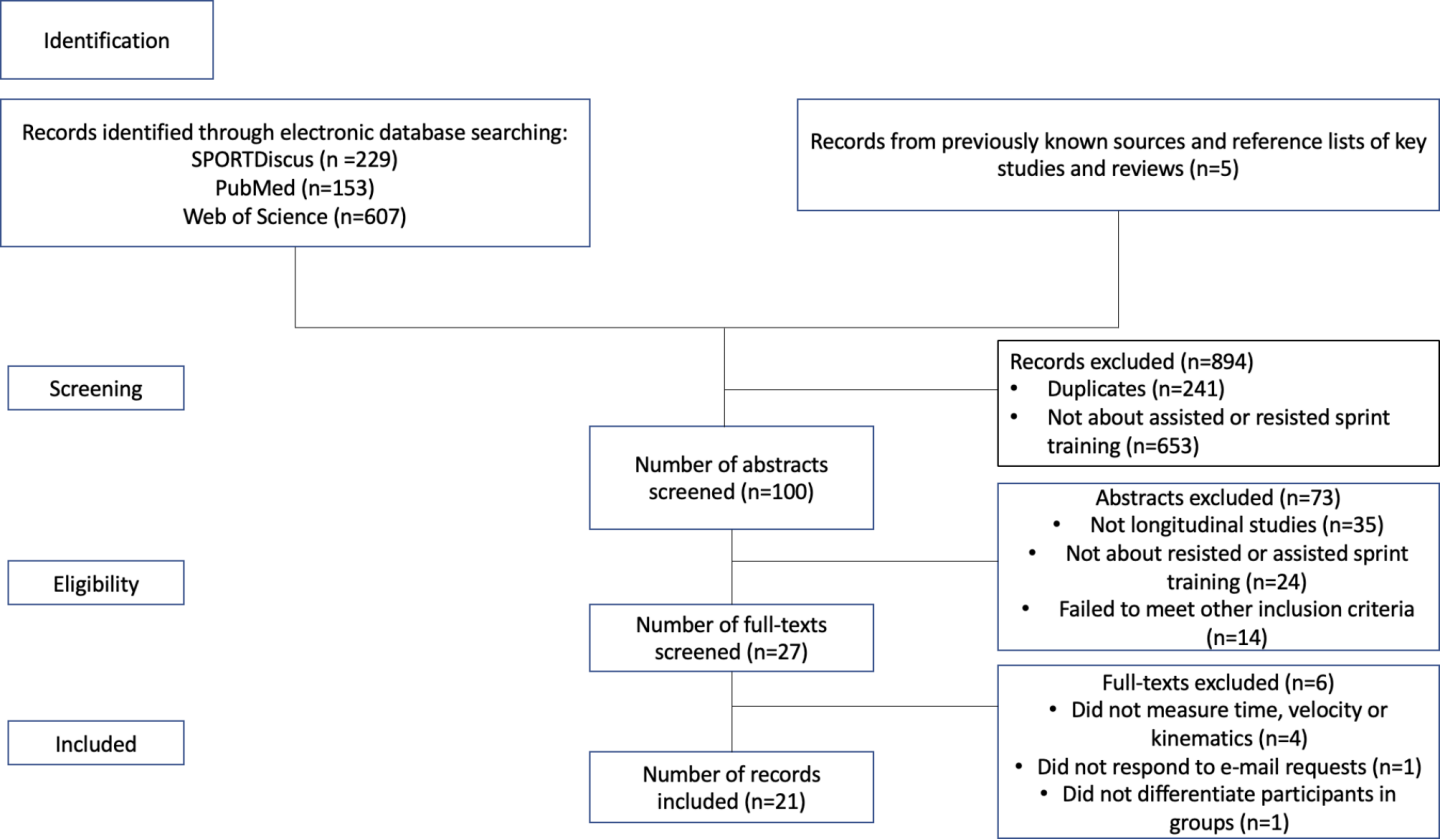
Flow of information through the systematic review process

A total of 994 studies were found following the study selection process, and after a thorough inclusion and exclusion process, 21 studies were included in this meta-analysis (Table 3; Fig. 8). Only 10 of the included studies measured step frequency, step length, and ground contact time. Only 9 of the studies measured flight time, 10 measured maximum velocity, and 9 studies measured 10-m times. Seven studies measured 20-m times, and only 4 studies measured 30-m times. Therefore, it was decided not to include the 30-m time for further meta-analysis. The surfaces used in the studies were track (9), grass (1), hard surface (9), and a soccer field (1). The time assessment methods used in the studies were photocells (14), stopwatch (1), radar device (4), and another method to record the sprint performance (1). The studies that measured kinematics (10) used some form of high-speed camera to investigate the changes (Table 1).

**Table 3:** Types of intervention studies, duration, number of subjects, and their anthropometrics in alphabetic order

Study	Load type	CG (*n*)	IG (*n*)	Duration (weeks)	Males / Females	Age (Years)	Weight (kg)	Height (cm)	Level
Bachero-Mena et al. [34]	Resisted sled low load	0	7	7 weeks	m	21.9 ± 2.3	75.8 ± 10.7	180.9 ± 6.78	Recreationally active
	Resisted sled medium load	0	6		m	20.8 ± 2.0	66.8 ± 8.5	173.8 ± 4.58	
	Resisted sled high load	0	6		m	19.8 ± 1.6	70.2 ± 11.9	175.4 ± 6.79	
Cahill et al. [36]	Resisted sled pull	12	53	8 weeks	m	16.9 ± 0.8	76.4 ± 13.6	175 ± 7.1	Highly trained
Cahill et al. [37]	Resisted sled push	12	50	8 weeks	m	16.6 ± 0.8	74.3 ± 11.5	175 ± 7.1	Highly trained
Clark et al. [38]	Resisted sled	7	7	7 weeks	m	19.7 ± 1.0	87.9 ± 17.3	181.15 ± 6.8	Trained
	Resisted vest	7	6			19.8 ± 0.9	79.15 ± 0.9	182.25 ± 8.36	
Hicks et al. [40]	Assisted	8	14	7 weeks	m	14.4 ± 0.3	58.5 ± 10	174 ± 0.08	Trained
Kristensen et al. [15]	Assisted Resisted	7 7	6 6	6 weeks	f + m	22 ± 2.6	73.8 ± 8.8	176 ± 0.1	Recreationally active
Lockie et al. [41]	Resisted sled	9	9	6 weeks	m	23.1 ± 4.2	83.1 ± 8.6	182 ± 0.1	Trained
Luteberget et al. [4]	Resisted sled	9	9	10 weeks	f	20.4 ± 3.1	74.6 ± 5.9	170.3 ± 5.3	Highly trained
Makaruk et al. [42]	Assisted	12	12	5 weeks	f	21.5 ± 0.8	63.3 ± 6.4	170 ± 0.06	Recreationally active
Martinopoulou et al. [42]	Resisted parachute	8	8	4 weeks	m	25 ± 4	61.5 ± 10.2	172 ± 0.8	Highly trained
Morin et al. [21]	Resisted sled	6	10	8 weeks	m	26.3 ± 4	74.5 ± 5.3	177 ± 0.08	Highly trained
Paradisis et al. [44]	Uphill + downhill, uphill, downhill	7	21	6 weeks	m	23.8 ± 1.4	75.9 ± 9.5	176 ± 0.07	Trained
Paradisis et al. [11]	Uphill-downhill	18	18	8 Weeks	m	24.1 ± 2.1	75.3 ± 10.2	175 ± 0.08	Trained
Paradisis et al. [30]	Uphill-downhill	6	6	6 weeks	f + m	25.3 ± 2.9	71.2 ± 9.4	175 ± 0.06	Highly trained
Pareja-Blanco et al. [44]	Resisted	14	14	8 weeks	f	22.0 ± 1.5	58.6 ± 10.4	161 ± 0.08	Trained
	Control		14		f	22.0 ± 1.5	58.7 ± 5.7	162 ± 0.04	
Rodríguez-Rosell et al. [45]	Resisted sled								Trained
	20%	12	12	8 weeks	m	23.8 ± 2.2	77.9 ± 10.2	179 ± 0.08	
	40%		12		m	22.1 ± 1.7	72.5 ± 3.2	177 ± 0.05	
	60%		12		m	21.9 ± 2.2	75.3 ± 10.4	178 ± 0.09	
	80%		12		m	22.2 ± 1.8	72.4 ± 8.6	176 ± 0.09	
Sinclair et al. [46]	Resisted 20% velocity decrement	14	14	8 weeks	m	18.8 ± 0.6	87.6 ± 11.4	182.2 ± 5.5	Highly trained
Spinks et al. [22]	Resisted sled	10	10	8 weeks	m	21.8 ± 4,2	83.3 ± 8.7	181.9 ± 6.2	Highly trained
Upton [23]	Resisted towing system	9	9	4 weeks	f	19.6 ± 0.9	63.4 ± 6.9	166.9 ± 5.9	Highly trained
	Assisted	9	9		f				
West et al. [47]	Resisted sled	10	10	6 weeks	m	26.8 ± 3.0	90.2 ± 10.3	186 ± 8.0	Highly trained
Zafeiridis et al. [48]	Resisted sled	11	11	8 weeks	m	20.1 ± 1.9	73.1 ± 2.4	178.0 ± 7.0	Recreationally active

### Evaluation of Potential Publication Bias

As suggested by Higgins et al. [35] there should be at least 10 studies in a meta-analysis subgroup to accurately perform a trim and fill. As only 10-m, 20-m and maximum velocity analysis met these requirements, the funnel plots for these three measures were the only ones assessed. The 10-m funnel plot was symmetrical, while the 20-m and maximal velocity funnel plots for the resisted sprint studies were not. Therefore, a trim and fill was performed for these results. The trim and fill results suggested that three studies might be missing for the 20-m resisted sprint analysis, and one study is missing for the maximum velocity analysis. After adjusting for publication bias, the overall effect size was 0.09 for the 20-m analysis and 0.06 for the maximum velocity analysis.

### Sprint Kinematics

The changes that resisted and assisted sprint training induced on sprint kinematics were measured in 108 participants for step frequency and step length. For ground contact time and flight time, 87 participants were tested.

No significant effect between intervention and control groups on step length was found when all the studies were included (Z = 1.08, *P* = 0.28), or when comparing intervention to control groups for the resisted, assisted, and combined uphill and downhill subgroups (Z = 1.00, *P* = 0.32; Z = 1.3, *P* = 0.19; Z = 0.35, *P* = 0.73, Fig. 1). Furthermore, no significant effect between intervention and control groups on contact time was found when all studies were included (Z = 0,20, *P* = 0.84), or for the resisted, assisted, and combined uphill and downhill subgroups (Z = 0.11, *P* = 0.91; Z = 0.41, *P* = 0.68; Z = 0.12, *P* = 0.91) (Fig. 2). A moderate effect size of 0.6 on contact time, and a moderate effect size on step frequency of 0.67 for assisted sprint training,were found (Table 2).

No significant effects between intervention and control group on flight time were found when evaluated for all studies included (Z = 0.85, *P* = 0.39) (Fig. 3), or when evaluating resisted, assisted, and combined uphill and downhill subgroups (Z = 1.09, *P* = 0.27; Z = 0.05, *P* = 0.96; Z = 0.21, *P* = 0.84) (Fig. 3). Combined effect sizes for flight times were small for all categories (Table 2).

There was no significant effect on changes in step frequency between intervention and control groups when evaluated for all studies included (Z = 1.75, *P* = 0.08), or in the resisted group, assisted and uphill–downhill subgroups (Z = 0.78, *P* = 0.44; Z = 0.66, *P* = 0.51; Z = 1.63, *P* = 0.51, (Fig. 4). Combined effect sizes for step frequency were small for resisted and combined training, while moderate for assisted sprinting (Table 2).

### Acceleration Phase

The effects of resisted and assisted sprint training on acceleration were measured in 198 participants for the 10-m time and 181 participants for the 20-m time. Unfortunately, the analysis of the 30-m time had too few studies to be included. When comparing the pre-test to the post-test between the intervention groups and the control group, the intervention group had a significantly greater decrease in 10-m times compared to the control group (Z = 2.01, *P* = 0.04). The 95% CI (0.22 to 0.43) indicated a small effect in favor of the intervention group (Fig. 6). There were no significant effects in the assisted sprint group (Z = 1.74, *P* = 0.08).

For the 20-m times, the analysis showed no significant difference for either the resisted or assisted groups when comparing the intervention and control groups (Z = 1.45, *P* = 0.15; Z = 1.18, *P* = 0.24). However, the 95% CI (0.16 to 0.38) favoured the intervention group (Fig. 7). Moderate effect sizes were found for resisted sprint training (0.55 on 10-m times) and assisted sprint training (0.73 on 10-m times and 0.53 on 20-m times) (Table 2).

### Maximum Velocity

The effects of resisted, assisted and combined uphill-downhill sprint training on maximum velocity were measured in 186 participants. No significant effects on changes in maximum velocity were found in either the analysis of all studies, or in the different subgroups of resisted, assisted and uphill-downhill (Z = 0.06, *P* = 0.95; Z = 0.7, *P* = 0.48; Z = 0.09, *P* = 0.93; Z = 1.4, *P* = 0.16, Fig. 5). Combined uphill-downhill sprint training found a moderate effect size on maximum velocity of 0.54 (Table 2).

## Discussion

The main objective of this systematic review with meta-analysis was to investigate the longitudinal effects of resisted training, assisted training, or a combination of both on sprint kinematics, acceleration, and maximum velocity. The main findings were a significant improvement in 10-m times for the resisted sprint training group compared to the control group (Fig. 6.). No other significant effects in the between groups analysis were found. However, within group effects showed that some of the different training methods had a moderate effect (Table 2). This included a moderate effect on increased maximum velocity using combined uphill-downhill sprint training, a moderate effect on decreased 10-m times using both resisted and assisted sprint training, a moderate effect on decreased 20-m times using assisted sprint training and a moderate effect on decreased contact time using assisted sprint training (Table 2).

Resisted sprint training is the method investigated most often within the literature among the different sprint training approaches, as demonstrated by the large number of studies (19) compared to assisted sprint training (6) and combined studies (3). It is difficult to conclude if the assisted or uphill–downhill method is superior to resisted sprint training based on the available evidence. However, the results might encourage more research in these areas, as some of the results were significant and there were considerable effect estimates within groups.

Some results showed a significant effect, while others did not. It is important to recognize that sprinting at a senior level is all about improving minimal margins each season [49]. The ESs observed in some of the significant findings were small, suggesting that the enhancements are minimal but still present. Therefore, conducting further research based on these findings may be of interest, as even marginal improvements can have a significant impact on high-end competitive sprinting. Consideration of the World Athletics Championships results in 2023 for the 100-m event shows that only milliseconds separate 2nd place from 4th [50] proving how marginal the sprint events are and why marginal improvements are still important. Although most of the kinematic changes in the resisted sprint training groups were not significant and exhibited considerable variation in ESs, they still show potential.

Several studies included in the analysis involved team sport athletes, such as handball, football, and rugby, which prioritize various attributes in their training. The results might have differed if all the studies exclusively focused on pure sprinters, as sprinting is their primary attribute. Sprinters also engage in heavy resistance training combined with plyometrics, which contributes to their sprint performance and could be a confounding variable in the analyzed results [51, 52].

### Acceleration

Strength training plays a key role in improving sprint performance [53]. During the acceleration phase, speed development depends mainly on powerful extensions of all leg joints. Faster acceleration requires the involvement of more muscle mass [54]. It would be expected that the additional resistance during resisted sprint training would be beneficial for improving sprint performance.

The 10-m times decreased in the overall group with training interventions. The group consisted of all but one study that trained with resistance and one study that used assistance. The ES of the training intervention were moderate, suggesting a noticeable effect on performance. When comparing the pre- to post-test results for the intervention group performing resisted or assisted sprints, the intervention group had a significantly greater decrease in 10-m times compared to the control group (Fig. 6). Hicks et al. [39] was the only assisted sprint training study that measured 10 and 20-m times. This study found a moderate effect (Table 2) which may indicate that assisted sprints may be beneficial for acceleration. However, this is difficult to conclude, and more research needs to be done on the subject.

To have effective acceleration, the step frequency should be as high as possible for the first few steps [55]. The aim is therefore to increase step frequency. None of the subgroups found a significant change in step frequency when compared to the control group (Fig. 4), but as an improvement in acceleration was found, it is possible that step frequency also was affected to some degree. However, the studies measuring kinematics were mostly different studies to the studies measuring acceleration times. Especially the assisted sprint study by Hicks et al. [39] used in the 10- and 20-m analyses which was not used in the analysis of the sprint kinematics, as it did not measure any of these parameters. Therefore, it is hard to conclude if the increased step frequency in the assisted groups caused faster acceleration because it was not the same study (Fig. 4).

Furthermore, none of the groups demonstrated a change in flight time. A change in flight time could potentially be a contributing factor to the observed acceleration improvement as the effect estimates were small to moderate in all the different experimental training groups (Table 2). With shorter flight times, step frequency tends to increase. Ito et al. [56] suggested that athletes should aim to increase the step frequency at the start, but also strive for an increased step length to improve their acceleration. However, if athletes aim to increase step frequency, step length will decrease [57]. Since step frequency is closely related to step length, flight time, and ground contact time [58, 59], the significant decrease in flight time observed in the overall group may have influenced step frequency or step length and, consequently, improved acceleration. The meta-analysis revealed no significant changes in ground contact time (Fig. 2). When considering individual studies on resisted sprint training, Lockie et al. [19] and Cronin et al. [18] indicated an increase in ground contact time during resisted sprint training, which is advantageous for the acceleration phase, providing more time to generate force at the start of the sprint. Also, Makaruk et al. [41] found an increase in contact time, but also a decrease in step frequency. Unfortunately, as the data from this study could not be obtained, the study was not included in the analysis, which could potentially have influenced the results. Conversely, Lockie et al. [40] found a significant increase in step length, and that increase equally improved acceleration.

Considering the significant improvement in the acceleration observed with resisted sprint training, this training method should also be applied to team sport athletes to improve overall performance. As high acceleration is crucial in the fast break in handball [5], a faster acceleration is also important in soccer, rugby, and field hockey [8–10]. However, coaches may keep in mind that training with resistance in sprint training increases the overall training loads. Luteberget et al. [4] showed that handball athletes in the middle of a season showed less improvement in the intervention group (resisted sprints) compared to the control group. This could be attributed to the excessive load in the intervention group, although this is speculative. Nonetheless, the results of the meta-analysis indicated that resisted sprint training improves acceleration more effectively than traditional sprint training.

Moreover, it is worth noting that sprinters may have a higher tolerance to training with extra loading compared to team sport athletes, which also prioritizes other characteristics. Comparisons between athlete subsets showed that sprinters exhibited a greater capacity for maximum horizontal power than recreational athletes, particularly when expressed relative to body mass [60]. This suggests that resisted sprint training may have a more pronounced effect on elite sprinters than on team sport athletes as this type of training is considered a type of training to develop specific strength [61]. As sprinters are already optimized for high-speed power output due to their pre-existing high level of neuromuscular adaptation to sprinting tasks, team sport athletes certainly have room for improvement in force production along the horizontal axis. Their diverse training might mean that the relative gains from resisted sprints are distributed across multiple fitness domains rather than concentrated in force applied horizontally during sprinting. This is shown by a meta-analysis by Ward et al. [62], which found no difference in improvement between resisted sprint training and normal sprinting in field-based team sport athletes.

### Maximum Velocity

No significant changes in maximum velocity between the experimental group and control groups in any of the subgroups (Fig. 5) were found. But closer examination of the effect estimates of the within group data, reveals some interesting findings. The resisted group showed no significant change in maximum velocity, the results were scattered and the effect size of the within group analysis was close to zero. This suggests that resisted sprint training was no better than normal sprinting at improving maximum velocity; the different ESs suggest that the effects of resisted sprint training on maximum velocity are unclear and not conclusive (Table 2). When training with resistance, sprinters will either tow or pull a load, which slows down the sprinter, especially in the acceleration phase. While a weighted sled gains momentum and becomes lighter over time, the increased weight keeps the athlete in a crouched position for longer, emphasizing acceleration [63]. This may explain why the athletes perform better in the 10-m and 20-m measurements but experience close to no change in their maximum velocity. Additionally, training with resistance was associated with an increased trunk lean, which allows for a greater application of force in the horizontal direction but is not ideal for the maximal velocity phase of sprinting [63].

On the other hand, the assisted sprint training group did not exhibit a significant improvement in maximum velocity either (Fig. 5). However, there are different forms that create the assisted sprint condition. Makaruk et al. [41] used a towing system for assistance, while Paradisis et al. [43] used a downhill approach. Both studies reported a significant increase in maximum velocity, highlighting the potential benefits of assisted sprint training. When training assisted sprints, athletes can achieve higher speeds than their normal maximum velocity. The effects of these different forms of overspeed stimulation may vary but suggest an increase in maximum velocity after training with assistance, but due to the limited number of studies examining the longitudinal effects of assisted sprint training, the question remains unanswered.

An interesting finding concerning the longitudinal effects of assisted sprint training was that there was some variability observed with a decrease in step length (± 0.02 m) in the longitudinal studies (Fig. 1). This is the opposite of the acute studies that showed an increase in step length [20, 24, 25]. This may only be a coincidence due to the limited number of two studies. However, there were significant findings that assisted sprint training increased the step frequency as there was a moderate within group effect found (Table 2), likely a result of decreased contact and flight time (Figs. 2 and 3) [41, 43]. It seems that the pulling force gives a stimulus over time to move the joints faster during sprinting to avoid too much braking or being too late for the next step [25]. This could compensate for the decrease in step length. This may explain an increase in maximum velocity.

Regarding the combined group, there was no significant change in maximum velocity (Fig. 5), although a moderate effect size in the within group analysis was observed (Table 2). Paradisis et al. [43] indicated that uphill–downhill training could be beneficial for improving both acceleration and maximum velocity. The combined uphill–downhill group exhibited the greatest increase in overall sprint performance, with significant improvements in maximum velocity, step frequency, and ground contact time. However, there are only three studies on this topic, and they were all performed by the same research group [11, 30]. This should be investigated further by other research groups. The result of the meta-analysis suggests that this is potentially an effective form of training for sprinters or team sport athletes, but it is difficult to provide the set-up described in these studies. As a result, this form of training may be less relevant to practical application.

### Kinematic Changes

When only investigating the kinematic changes during the different interventions, the results were varied. None of the groups had any significant changes compared to the control group (Figs. 1, 2, 3 and 4). However, using resisted sprint training alone did not lead to significant changes in sprint kinematics. This is strange, considering resisted sprint training showed an increased improvement in the early acceleration phase of the run (Figs. 6 and 7). The findings for assisted sprint training align with previous acute studies conducted on assisted sprint training, which found an increase in step frequency [20, 24, 25], thereby indicating that the acute effect can become a longitudinal effect for this training approach. The ESs were moderate in both contact time and step frequency. In the combined uphill–downhill group, the changes were non-significant with a small ES. Although the ES was small, which suggests minimal improvement, considering the narrow margins in elite sprinting, it is worth further research to investigate the effects of uphill–downhill training on step frequency (Fig. 4).

### Limitations

It is important to note that this review has some limitations that should be considered when interpreting the results. Firstly, the sample size was relatively small, especially when analyzing subgroups, which may limit the generalizability of the findings. Secondly, the study only measured the short-term effects of the interventions, so it is unclear whether these improvements would be maintained over a longer period. Thirdly, there is probably a publication bias, which means that when an intervention has positive results, it is more likely that it is published than when it does not have any effect upon sprinting results. Fourthly, the different loading schemes were not considered in this review and meta-analysis. Therefore, it should be acknowledged that different loads may lead to different acute effects and adaptations. Finally, the review did not measure any other potential factors that may influence sprint performance, such as strength or flexibility.

## Conclusion

When comparing the experimental groups with the control groups performing normal sprinting, none of the training methods proved to be more effective than normal sprinting in altering kinematics or improving maximum velocity. Using resisted sprints resulted in a significant increase in 10-m performance compared to normal sprinting, but no significant interaction was found for 20-m. For the within group analysis presented as effect size, it is concluded that combined (uphill–downhill) training exhibited the greatest increase in overall sprint performance, with improvements in maximum velocity, due to higher step frequency, caused by shorter flight times. Assisted sprint training may be more effective for improving step frequency, and it seems that resisted sprints are effective for improving acceleration ability (10–20 m), but not maximum velocity. However, coaches and athletes should consider the specific kinematic measures they want to improve and choose the most appropriate training protocol based on what they are seeking to improve.

## Electronic Supplementary Material

Below is the link to the electronic supplementary material.

Supplementary Material 1

## Data Availability

Please contact the corresponding author for data requests.

## Abbreviations

BW: Body weight
CG: Control group
CI: Confidence interval
ES: Effect size
F: Female
Hz: Hertz
IG: Intervention group
M: Male
Wk: Week
s: Seconds
Hz: Hertz
M/s: Meters divided by seconds

## References

[1] Mu-Hui Y, Chiu-Chou C, Wei-Hua HO, Ching-Ting HSU. The relationship between velocity utilization rate and Pole vault performance. J Hum Sport Exerc. 2021;16(2):273–83.

[2] Murakami M, Tanabe S, Ishikawa M, Ito A. The relationship between approach run kinematics and javelin throwing performance. Asian J Coaching Sci. 2017;1(1):1–14.

[3] Abd Rahim MA, Lee ELY, Abd Malek NF, Suwankhong D, Nadzalan AM. Relationship between physical fitness and long jump performance. Age (Years old). 2020;21:271.

[4] Luteberget LS, Raastad T, Seynnes O, Spencer M. Effect of traditional and resisted sprint training in highly trained female team handball players. Int J Sports Physiol Perform. 2015;10(5):642–7. 25569506 10.1123/ijspp.2014-0276

[5] Šbila M, Vuleta D, Pori P. Position-related differences in volume and intensity of large-scale cyclic movements of male players in handball. Kinesiology. 2004;36(1):58–68.

[6] TaSkin H. Evaluating sprinting ability, density of acceleration, and speed dribbling ability of professional soccer players with respect to their positions. J Strength Conditioning Res. 2008;22(5):1481–6. 10.1519/JSC.0b013e318181fd90 18714240

[7] Baker D, Nance S. The relation between running speed and measures of strength and power in professional rugby league players. J Strength Conditioning Res. 1999;13(3):230–5.

[8] Rumpf MC, Lockie RG, Cronin JB, Jalilvand F. Effect of different sprint training methods on sprint performance over various distances: a brief review. J Strength Conditioning Res. 2016;30(6):1767–85. 10.1519/JSC.0000000000001245 26492101

[9] Faude O, Koch T, Meyer T. Straight sprinting is the most frequent action in goal situations in professional football. J Sports Sci. 2012;30(7):625–31. 22394328 10.1080/02640414.2012.665940

[10] Rienzi E, Drust B, Reilly T, Carter JEL, Martin A. Investigation of anthropometric and work-rate profiles of elite south American international soccer players. J Sports Med Phys Fitness. 2000;40(2):162. 11034438

[11] Paradisis GP, Bissas A, Cooke CB. Combined uphill and downhill sprint running training is more efficacious than horizontal. Int J Sports Physiol Perform. 2009;4(2):229–43. 19567926 10.1123/ijspp.4.2.229

[12] Hughes W, Healy R, Lyons M, Nevill A, Higginbotham C, Lane A, et al. The effect of different strength training modalities on sprint performance in female team-sport athletes: a systematic review and meta-analysis. Sports Med. 2023;53(5):993–1015. 36877405 10.1007/s40279-023-01820-5

[13] Suarez DG, Wagle JP, Cunanan AJ, Sausaman RW, Stone MH. Dynamic correspondence of resistance training to sport: a brief review. Strength Conditioning J. 2019;41(4):80–8.

[14] van den Tillaar R, Gamble P. Comparison of step-by‐step kinematics and muscle activation of resisted, assisted, and unloaded 30‐m sprints in sprinters. Translational Sports Med. 2018;1(4):151–9.

[15] Kristensen GO, Van den Tillaar R, Ettema GJ. Velocity specificity in early-phase sprint training. J Strength Conditioning Res. 2006;20(4):833–7. 10.1519/R-17805.1 17194234

[16] de Villarreal ES, Requena B, Cronin JB. The effects of plyometric training on sprint performance: a meta-analysis. J Strength Conditioning Res. 2012;26(2):575–84. 10.1519/JSC.0b013e318220fd03 22240550

[17] Haugen T, Seiler S, Sandbakk Ø, Tønnessen E. The training and development of elite sprint performance: an integration of scientific and best practice literature. Sports Medicine-Open. 2019;5:1–16. 31754845 10.1186/s40798-019-0221-0 PMC6872694

[18] Cronin J, Hansen KT. Resisted sprint training for the acceleration phase of sprinting. Strength Conditioning J. 2006;28(4):42–51.

[19] Lockie RG, Murphy AJ, Spinks CD. Effects of resisted sled towing on sprint kinematics in field-sport athletes. J Strength Conditioning Res. 2003;17(4):760–7. 10.1519/1533-4287(2003)017<0760:eorsto>2.0.co;2 14636109

[20] van den Tillaar R, von Heimburg E. Comparison of different sprint training sessions with assisted and resisted running: effects on performance and kinematics in 20-m sprints. Hum Mov. 2017;18(2):21–9.

[21] Morin J-B, Petrakos G, Jiménez-Reyes P, Brown SR, Samozino P, Cross MR. Very-heavy sled training for improving horizontal-force output in soccer players. Int J Sports Physiol Perform. 2017;12(6):840–4. 27834560 10.1123/ijspp.2016-0444

[22] Spinks CD, Murphy AJ, Spinks WL, Lockie RG. The effects of resisted sprint training on acceleration performance and kinematics in soccer, rugby union and Australian football players. J Strength Conditioning Res. 2007;21(1):77–85. 10.1519/00124278-200702000-00015 17313259

[23] Upton DE. The effect of assisted and resisted sprint training on acceleration and velocity in Division IA female soccer athletes. J Strength Conditioning Res. 2011;25(10):2645–52. 10.1519/JSC.0b013e318201be16 21873906

[24] van den Tillaar R, Gamble P. Comparison of step-by-step kinematics of resisted, assisted and unloaded 20-m sprint runs. Sports Biomech. 2019;18(5):539–52. 29578385 10.1080/14763141.2018.1442871

[25] Cecilia-Gallego P, Odriozola A, Beltran-Garrido JV, Álvarez-Herms J. Acute effects of overspeed stimuli with towing system on athletic sprint performance: a systematic review with meta-analysis. J Sports Sci. 2022;40(6):704–16. 34991419 10.1080/02640414.2021.2015165

[26] Mann R, Murphy A. The mechanics of sprinting and hurdling: 2013 edition. Lexington, KY2013. 2013.

[27] Economou T, Stavridis I, Zisi M, Fragkoulis E, Olanemi-Agilara G, Paradisis G. Sprint mechanical and kinematic characteristics of national female track and field champions and lower-level competitors. J Phys Educ Sport. 2021;21:3227–35.

[28] Hicks D. Resisted and assisted sprint training: determining the transfer to maximal sprinting. New Stud Athletics. 2018;10/19:32:35–51.

[29] Osterwald KM, Kelly DT, Comyns TM, Catháin CÓ. Resisted sled sprint kinematics: the acute effect of load and sporting population. Sports (2075–4663). 2021;9(10):137. 34678918 10.3390/sports9100137 PMC8538495

[30] Paradisis GP, Bissas A, Cooke CB. Effect of combined uphill-downhill sprint training on kinematics and maximum running speed in experienced sprinters. Int J Sports Sci Coaching. 2015;10(5):887–97.

[31] Moher D, Liberati A, Tetzlaff J, Altman DG. PRISMA Group* t. Preferred reporting items for systematic reviews and meta-analyses: the PRISMA statement. Ann Intern Med. 2009;151(4):264–9. 19622511 10.7326/0003-4819-151-4-200908180-00135

[32] Bachero-Mena B, GonzÁLez-Badillo JJ. Effects of resisted sprint trainingon acceleration with three different loads accounting for 5, 12.5, and 20% of body mass. J Strength Conditioning Res. 2014;28(10):2954–60. 10.1519/JSC.0000000000000492 24736770

[33] Bhogal SK, Teasell RW, Foley NC, Speechley MR. The PEDro scale provides a more comprehensive measure of methodological quality than the Jadad scale in stroke rehabilitation literature. J Clin Epidemiol. 2005;58(7):668–73. 15939217 10.1016/j.jclinepi.2005.01.002

[34] Cohen J. Statistical power analysis for the behavioral sciences. Academic; 2013.

[35] Higgins JP, Green S. Cochrane handbook for systematic reviews of interventions. 2008.

[36] Cahill MJ, Oliver JL, Cronin JB, Clark K, Cross MR, Lloyd RS, et al. Influence of resisted sled-pull training on the sprint force-velocity profile of male high-school athletes. J Strength Conditioning Res. 2020;34(10):2751–9. 10.1519/JSC.0000000000003770 32773545

[37] Cahill MJ, Oliver JL, Cronin JB, Clark KP, Cross MR, Lloyd RS. Influence of resisted sled-push training on the sprint force‐velocity profile of male high school athletes. Scand J Med Sci Sports. 2020;30(3):442–9. 31742795 10.1111/sms.13600

[38] Clark KR, Stearne DJ, Walts CT, Miller AD. The longitudinal effects of resisted sprint training using weightes sleds vs. weighted vests. J Strength Conditioning Res. 2010;24(12):3287–95. 10.1519/JSC.0b013e3181b62c0a 19996786

[39] Hicks DS, Drummond C, Williams KJ, van den Tillaar R. The effect of a combined sprint training intervention on sprint force-velocity characteristics in junior Australian football players. PeerJ. 2023;11:e14873. 36941999 10.7717/peerj.14873 PMC10024483

[40] Lockie RG, Murphy AJ, Schultz AB, Knight TJ, Janse De Jonge XAK. The effects of different speed training protocols on sprint acceleration kinematics and muscle strength and power in field sport athletes. J Strength Conditioning Res. 2012;26(6):1539–50. 10.1519/JSC.0b013e318234e8a0 21912294

[41] Makaruk B, Sozanski H, Makaruk H, Sacewicz T. The effects of resisted sprint training on speed performance in women. Hum Mov. 2013;14(2):116–22.

[42] Martinopoulou K, Polyxeni A, Paradisis G, Katsikas C, Smirniotoy A. The effects of resisted training using parachute on sprint performance. J Biology Exerc. 2011 01/01;7:7.

[43] Paradisis GP, Cooke CB. The effects of sprint running training on sloping surfaces. J Strength Conditioning Res. 2006;20(4):767–77. 10.1519/R-16834.1 17194229

[44] Pareja-Blanco F, Sáez de Villarreal E, Bachero-Mena B, Mora-Custodio R, Asián-Clemente JA, Loturco I, et al. Effects of unloaded sprint and heavy sled training on sprint performance in physically active women. Int J Sports Physiol Perform. 2020;15(10):1356–62. 33004682 10.1123/ijspp.2019-0862

[45] Rodríguez-Rosell D, Sáez de Villarreal E, Mora-Custodio R, Asián-Clemente JA, Bachero-Mena B, Loturco I, et al. Effects of different loading conditions during resisted sprint training on sprint performance. J Strength Conditioning Res. 2022;36(10):2725–32. 10.1519/JSC.0000000000003898 33337706

[46] Sinclair J, Edmundson CJ, Metcalfe J, Bottoms L, Atkins S, Bentley I. The effects of Sprint vs. resisted sled-based training; an 8-Week in-season Randomized Control intervention in Elite Rugby League players. Int J Environ Res Public Health. 2021;18(17). 10.3390/ijerph18179241 PMC8431106 34501831

[47] West DJ, Cunningham DJ, Bracken RM, Bevan HR, Crewther BT, Cook CJ, et al. Effects of resisted sprint training on acceleration in professional rugby union players. J Strength Conditioning Res. 2013;27(4):1014–8. 10.1519/JSC.0b013e3182606cff 22692118

[48] Zafeiridis A, Saraslanidis P, Manou V, Ioakimidis P, Dipla K, Kellis S. The effects of resisted sled-pulling sprint training on acceleration and maximum speed performance. J Sports Med Phys Fit. 2005;45(3):284–90. 16230978

[49] Boccia G, Cardinale M, Brustio PR. World-class sprinters’ careers: early success does not guarantee success at adult age. Int J Sports Physiol Perform 2021 01 Mar. 2021;16(3):367–74. 10.1123/ijspp.2020-0090 33296871

[50] World Athletics W, Championships B. 2023. 2023 [cited 2024 28/07–24]; https://worldathletics.org/results/world-athletics-championships/2023/world-athletics-championships-budapest-2023-7138987

[51] McBride JM, Blow D, Kirby TJ, Haines TL, Dayne AM, Triplett NT. Relationship between maximal squat strength and five, ten, and forty yard sprint times. J Strength Conditioning Res. 2009;23(6):1633–6. 10.1519/JSC.0b013e3181b2b8aa 19675504

[52] Markström JL, Olsson C-J. Countermovement jump peak force relative to body weight and jump height as predictors for sprint running performances:(in) homogeneity of track and field athletes? J Strength Conditioning Res. 2013;27(4):944–53. 10.1519/JSC.0b013e318260edad 22692108

[53] Delecluse C. Influence of strength training on sprint running performance. Current findings and implications for training. Sports Med. 1997;24(3):147–56. 9327528 10.2165/00007256-199724030-00001

[54] Delecluse C. Influence of strength training on sprint running performance: current findings and implications for training. Sports Med. 1997;24:147–56. 9327528 10.2165/00007256-199724030-00001

[55] Nagahara R, Naito H, Morin J-B, Zushi K. Association of acceleration with spatiotemporal variables in maximal sprinting. Int J Sports Med. 2014;35(09):755–61. 24577864 10.1055/s-0033-1363252

[56] Ito A, Ishikawa M, Isolehto J, Komi PV. Changes in the step width, step length, and step frequency of the world’s top sprinters during the 100 metres. New Stud Athletics. 2006;21(3):35.

[57] Monte A, Muollo V, Nardello F, Zamparo P. Sprint running: how changes in step frequency affect running mechanics and leg spring behaviour at maximal speed. J Sports Sci. 2017 2017/02/16;35(4):339–45. 10.1080/02640414.2016.1164336 27028346

[58] Mero A, Komi P, Gregor R. Biomechanics of sprint running: a review. Sports Med. 1992;13:376–92. 1615256 10.2165/00007256-199213060-00002

[59] Bezodis I. Investigations of the step length-step frequency relationship in sprinting. applied implications for performance; 2012.

[60] Cross MR, Brughelli M, Samozino P, Brown SR, Morin J-B. Optimal loading for maximizing power during sled-resisted sprinting. Int J Sports Physiol Perform. 2017;12(8):1069–77. 28051333 10.1123/ijspp.2016-0362

[61] Verkhoshansky Y, Verkhoshansky N. Special strength training: manual for coaches. Verkhoshansky Sstm Rome; 2011.

[62] Ward C, Catháin CÓ, Chéilleachair NN, Grassick S, Kelly DT. Does resisted sprint training improve the sprint performance of field-based invasion team sport players? A systematic review and meta-analysis. Sports Med. 2024 2024/03/01;54(3):659–72. 10.1007/s40279-023-01952-8 37897636

[63] Petrakos G, Egan B, Morin J-B. Resisted sled sprint training to improve sprint performance: a systematic review. Sports Med. 2016;46(3):381–400. 26553497 10.1007/s40279-015-0422-8

