# Supplementary figures and images for: The Longitudinal Effects of Resisted and Assisted Sprint Training on Sprint Kinematics, Acceleration, and Maximum Velocity: A Systematic Review and Meta-analysis
Source: Sports Med Open. 2024 Oct 11;10:110. doi: 10.1186/s40798-024-00777-7 (PMC11469994; doi:10.1186/s40798-024-00777-7)

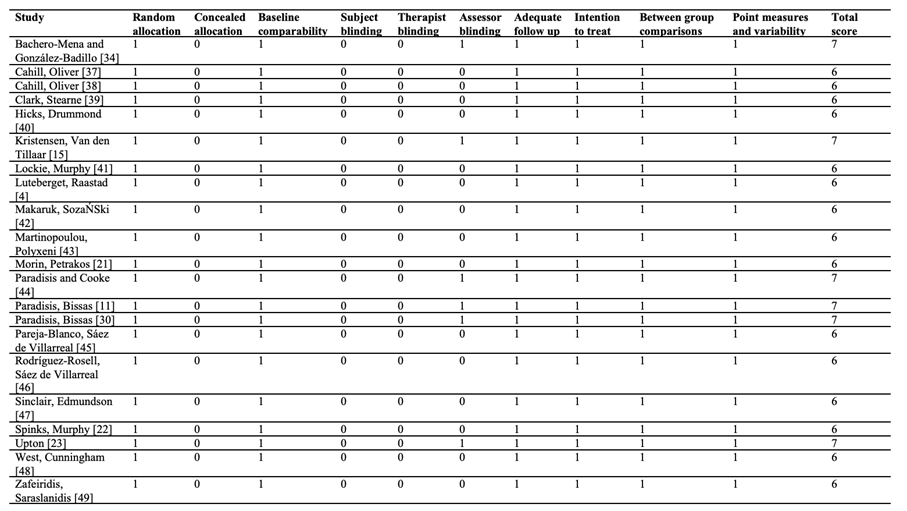
Pedro score of the included studies.

Supplement: Supplementary file 1 — Supplementary Material 1 [file 40798_2024_777_MOESM1_ESM.docx]
